# Annotations

**Published:** 1895-12-28

**Authors:** 


### Annotations.

### A Knavery Extirpation Society.

Diogenes took an infinity of trouble, with his keen eyes and his lantern, to search out an honest man. It is generally understood that he failed. Now the Greeks, of whom he was one, were the cleverest and the most intellectual people in the world at the time when Diogenes was their Thomas Carlyle. They were also the least honest. Moreover, Diogenes' own father though a banker, is said to have been one of the craftiest of a most dishonest race. Diogenes, therefore, became honest in sheer disgust at the dishonesty of his own family and fellow-countrymen. Something of this kind seems to be happening in our own country. Many of our fellow-countrymen, manufacturers and middlemen, have become such adepts at adulteration, cheating, and knavery, that a large number of people, in sheer cynical disgust, and without any appeal whatever to mere morality, have begun to say to one another that this kind of thing will not do any longer, and that a Diogenes' lantern must be made which will bring cheats and their deeds to light, and secure their punishment and their financial ruin. Here is one fact published by the modern many-headed Diogenes; and when that is considered, one feels that no more arguments are required. Dr. Thomas Stevenson, public analyst for the parish of St. Pancras, recently published his annual report, and in it he records that of 240 samples of different articles of food, beverages, and drugs analysed by him, no fewer than 78, or 1 in 3, were found to be adulterated. Let us understand fully what this means. It means, not that they were only finely or minutely adulterated, but grossly, largely; so grossly that a comparatively elementary analytical chemistry could aver for certain that they were dishonest articles. Are we to infer that the proportion of dishonest manufacturers and middlemen is the same as the proportions of their products; that is, 78 knaves to every 240 honest men? Probably we may! Most glad are we that a "Daniel has come to judgment" in the shape of the new "Anti-Adulteration Association." Will the association be able to do any real good? Decidedly it will if it loyally carries out its own programme. What it proposes to do is to analyse everybody's foods, beverages, and drugs, and to publish throughout the land not only the names of adulterated articles, but also the names of those who manufacture and of those who sell them. It is an excellent idea: Diogenes' lantern was not half so good. We wish the association, as the Irishman said, "more power to its elbow."

### The Chelsea Hospital for Women.

A special meeting of governors of this hospital was held on Wednesday, December 18th, at which, after some discussion, it was resolved "That the council, after full investigation and consideration, being of opinion that the continuance of Mr. O'Callaghan as a member of the medical staff would be incompatible with the maintenance of the proper discipline of the hospital, and injurious to its interests, and all the other members of the honorary medical staff having, in a formal manner, distinctively refused to co-operate with him in the discharge of their professional duties in the hospital, resolved that Mr. O'Callaghan be, and he is hereby, relieved of his duties as honorary medical officer of the hospital." We are of opinion, however, that the matter ought not to end here, and that there ought to be an inquiry by some trustworthy authority as to the events which have led to this unfortunate condition of affairs. It must be remembered that the board of management is a thoroughly discredited body, which ought to have resigned when its proceedings were impugned by the report of the committee of inquiry last year, and it is extremely unlikely that the public will accept their judgment in a case like this. What is clear is that the most absolutely opposed statements have been made on the two sides, and that until it is shown that truth lies on their side, the council lie under the stigma of having discharged a medical officer not because of any proved charge of inefficiency, but because, in the words of the resolution, the other members of the honorary medical staff "refused to co-operate with him in the discharge of their professional duties." Till the public know the truth they are sure to consider this a boycott.

### Charity and the Press.

How the Press can best help the community is a fairly large question, and it is easy to agree with the editor of the *Echo* that the conductors of each journal must decide for themselves what form should be taken by their service to humanity. Mr. Passmore Edwards, as he says, prefers founding institutions devoted to the permanent welfare of mankind to going round with the hat, soliciting subscriptions, and thereby trying to win a cheap popularity with other people's help. We have every sympathy with the principle, but cannot entirely agree with the not too graceful words in which it is put. Everyone will admire the long list of "Passmore Edwards" buildings, erected and promised, which is published in the *Echo*, and will be ready to recognise that the founder of these institutions has discovered a philanthropic and useful way of spending money, but few will agree with the sneer implied in the latter part of the sentence. As a wealthy man Mr. Passmore Edwards disposes of his wealth in a manner worthy of imitation. All honour to him! But then any rich man can if he chooses do the same thing. This, however, has nothing to do with the origin of his wealth, and is a very different thing from turning the very mode in which riches have been created into a mode of doing good. Journalism is a mighty power, endowed with infinite possibilities, and greatly as we admire Mr. Passmore Edwards and all his good works, we think that journalism is capable of something better than the mere making of money, even for good uses, and that when we see the daily work and labour of journalism lending itself to charity, organising good works, and even "passing round the hat," we cannot help feeling that in many cases even a better work is being done than when it becomes a mere money making process, however well the wealth produced may afterwards be spent.

